# An Uncommon Case of Syphilis With Simultaneous, Different Lesions: Moth-Eaten Alopecia, Syphilitic Keratoderma, and Psoriasiform Syphilis

**DOI:** 10.7759/cureus.49181

**Published:** 2023-11-21

**Authors:** Luca Pipitò, Manfredi Piccione, Marcello Trizzino, Cinzia Calà, Antonio Cascio

**Affiliations:** 1 Infectious and Tropical Diseases Unit; Department of Health Promotion, Mother and Child Care, Internal Medicine and Medical Specialties, Azienda Ospedaliera Universitaria Policlinico 'Paolo Giaccone', Palermo, ITA; 2 Microbiology and Virology Unit; Department of Health Promotion, Mother and Child Care, Internal Medicine and Medical Specialties, Azienda Ospedaliera Universitaria Policlinico 'Paolo Giaccone', Palermo, ITA

**Keywords:** moth-eaten pattern, secondary syphilis, atypical syphilis, atypical rash, syphilis

## Abstract

Syphilis is a re-emerging disease, and an increasing number of cases are being reported in Italy and worldwide. In this report, we present a case of a male patient with secondary syphilis characterized by the heterogenicity of the lesions: hyperkeratosis, psoriasiform-like lesions, papules, macules, and patchy alopecia on the scalp. The patient had applied several topical antimicrobials and steroid medicaments and taken oral acyclovir, which yielded no relief, for a previous wrong diagnosis. At the time of his presentation to our clinic, syphilis was suspected and confirmed by serology. The administration of a single intramuscular dose of penicillin led to a full recovery in three weeks. Screening for HIV and other sexually transmitted infections returned negative. Clinicians should maintain a high index of suspicion for syphilis when encountering sexually active patients with atypical skin manifestations.

## Introduction

Syphilis is a re-emerging sexually transmitted disease caused by the spirochete *Treponema pallidum*^ ^[1]. The primary infection may cause an ulcerated lesion (chancre) at the inoculation site, which is usually not painful, although atypical cases with painful, multiple, or different-looking lesions may occur. Although the primary lesions resolve even if the condition is not recognized and treated, the disease remains active [1]. In most cases, the disease evolves to the secondary stage within one to two months, which is characterized by a disseminated maculopapular rash [1]. However, atypical clinical pictures of secondary syphilis have been reported in the literature, and the skin lesions can have different aspects^ ^[2]. Ocular, cerebral, and other organ involvement may also occur [1]. Syphilis has historically been defined as "the great imitator". Not infrequently, these rashes are mistakenly diagnosed as other diseases and treated inappropriately, leading to the possibility that the infection is transmitted to other people and can evolve to the latency phase and eventually the final stage, the tertiary phase. The latter can involve the cardiovascular system, the central nervous system, and potentially any area with the formation of the gums [1]. We report an atypical case of secondary syphilis that was initially treated with topical antimicrobials, steroids, and acyclovir. The unique nature of our case was determined by the co-presence of lesions of varying appearance, which resembled other dermatological pathologies.

## Case presentation

A 40-year-old man presented to our clinic due to numerous diversified scattered asymptomatic skin lesions that had first appeared two months ago. He had applied several topical antimicrobials and steroid medicaments owing to misdiagnoses of infection and eczema, respectively. He had also taken oral acyclovir without any relief for the appearance of cheilitis, assuming it to be a sign of herpes labialis. However, he mentioned noticing an improvement in the condition on the trunk, characterized by a significant reduction of the lesions in the previous week, while a worsening of palmoplantar lesions had been observed. His medical history was otherwise unremarkable.

**Figure 1:**
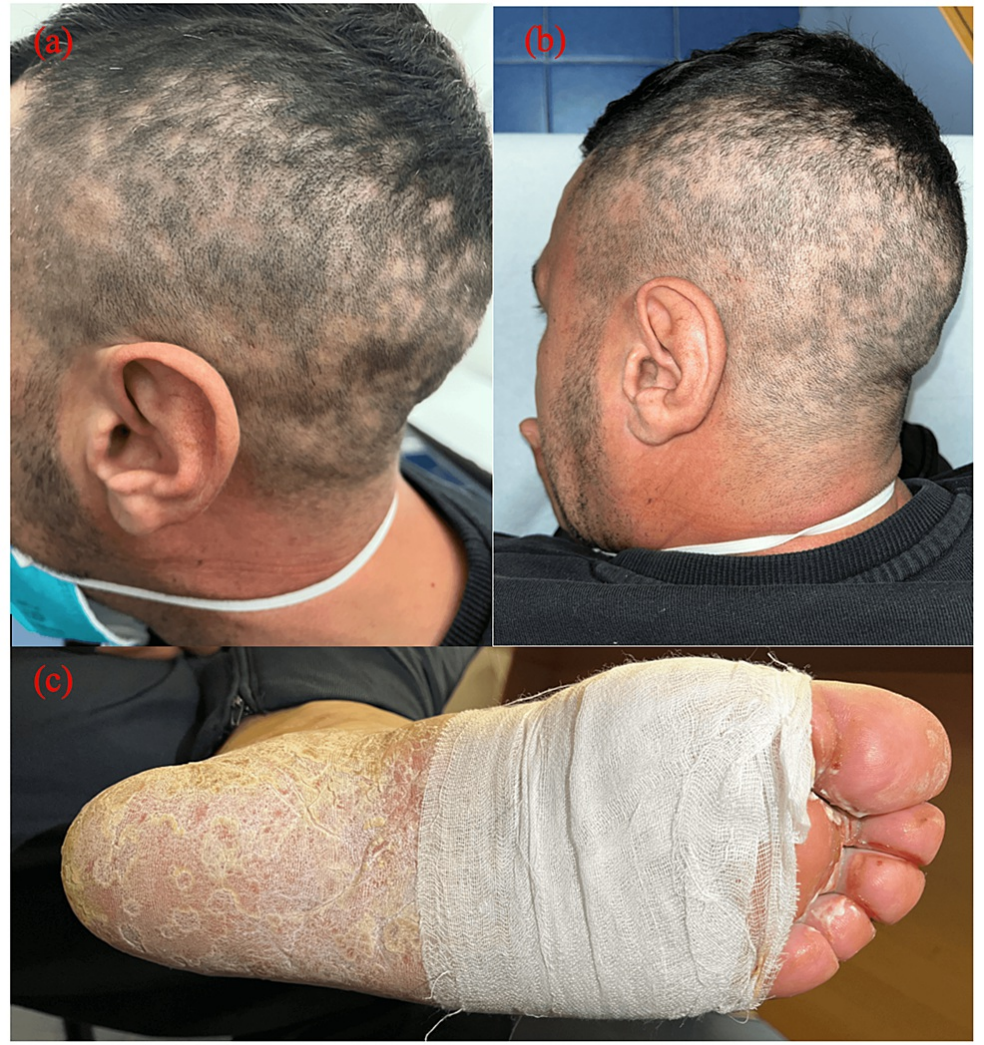
(a) and (b) Syphilis moth-eaten pattern characterized by patchy alopecia. (c) Syphilis keratoderma characterized by plantar hyperkeratosis

**Figure 2:**
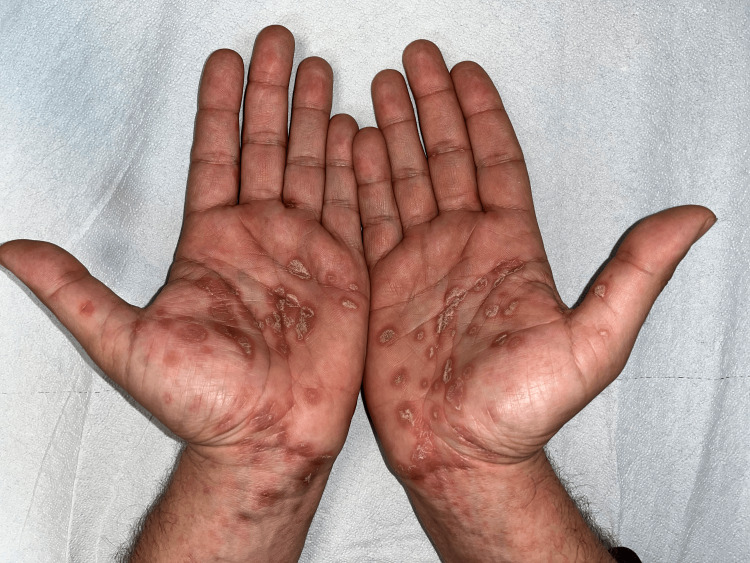
Desquamative papules of palms on erythematous bases

**Figure 3:**
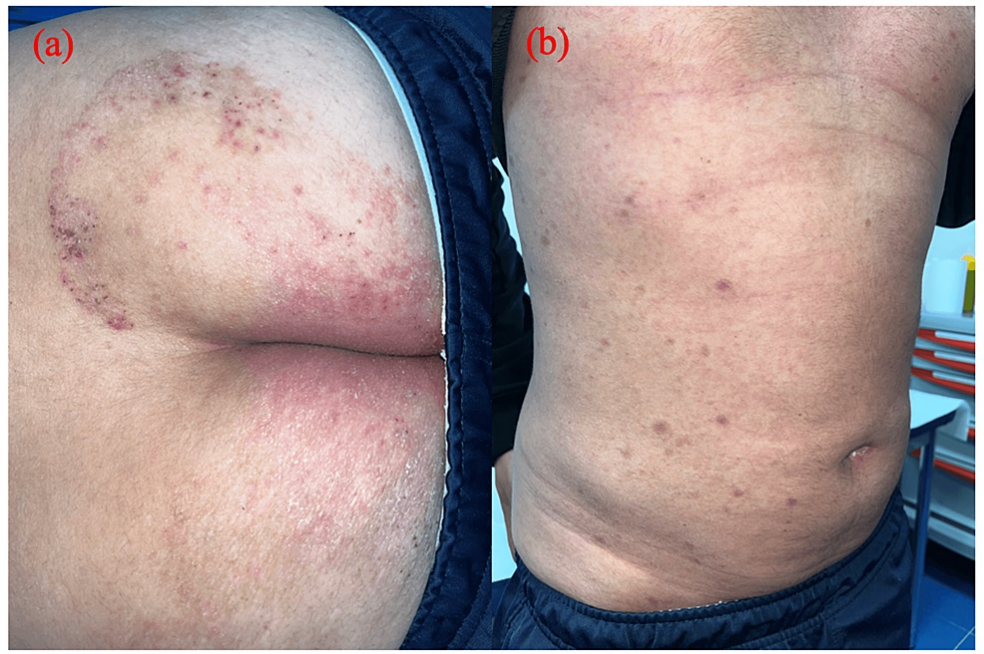
(a) Psoriasiform syphilis characterized by psoriasis-like lesions on the buttocks. (b) Nummular pigmented flat lesions on the trunk

At presentation, the patient was afebrile, and his vital parameters were unremarkable, with normal blood pressure as well as normal heart and respiratory rate. Physical examination showed several patterns of cutaneous manifestations. Non-scarring patchy alopecia was noted on the scalp (Figures 1a, 1b); angular cheilitis at the labial commissure, and desquamative papules on erythematous bases were observed on the palms (Figure 2); hyperkeratosis was highlighted on the plantar region of both feet, similar to an acquired keratoderma (Figure 1c), and intertriginous psoriasis-like lesions were observed on the buttocks (Figure 3a). Nummular pigmented flat lesions with thin peripheral scaling edges were scattered on the trunk (Figure 3b).

The patient's blood test revealed normal findings. He reported several instances of unprotected sexual intercourse with women. Treponemal test, rapid plasma reagin (RPR) for syphilis, serology for human HIV, hepatitis C virus (HCV), and hepatitis B virus (HBV), and molecular panel for sexually transmitted infections on the first urine flow (*Neisseria gonorrhoeae, Chlamydia trachomatis, Mycoplasma genitalium,* and *Trichomonas vaginalis*) were ordered. A diagnosis of secondary syphilis was suspected, and 2.4 MU of penicillin was administered intramuscularly.

Screening for HIV, HBV, HCV, and molecular panel for sexually transmitted infections was negative. However, the treponemal test and RPR (titer of 1:128) confirmed the diagnosis of syphilis. A rapid improvement was observed within a few days after the administration of intramuscular penicillin. The clinical picture resolved in three weeks, and all the skin lesions disappeared.

## Discussion

Syphilis is a re-emerging disease [1], and there has been a rise in the number of cases in Italy and worldwide. Our case highlighted the heterogenicity of the skin lesions associated with syphilis: hyperkeratosis, psoriasiform-like lesions, papules, and macules were observed in the same patient. It is no coincidence that syphilis is historically defined as "the great imitator". Secondary syphilis can involve any organ, and skin localizations can mimic numerous dermatologic and infectious diseases [2,3]. Primary lesions, usually asymptomatic and self-healing, may go unnoticed, and not infrequently, clinicians can be deceived by the presence of skin rash. Secondary syphilis may also be self-resolving, but unidentified infections lead to transmissions and the possible evolution towards tertiary forms [1].

In our case, the patchy alopecia and the palmar lesions suggested the diagnosis of syphilis, which was supported by the patient's history of several instances of unprotected sexual intercourse and eventually confirmed by serology. Non-scarring patchy alopecia is a hallmark known as a “moth-eaten pattern”. It may resemble a dermatophyte infection. It is caused by the invasion of spirochetes in the hair follicles and improves with treatment [4]. Its incidence rate in secondary syphilis ranges from 2.9 to 7% [5]. Plantar hyperkeratosis is the hallmark of syphilitic keratoderma. This form has rarely been described in the literature, and clinically it is indistinguishable from other common dermatoses on volar aspects, such as keratoderma blenorrhagicum, hyperkeratotic eczema, and keratoderma associated with Unna-Thost syndrome or Howel-Evans syndrome [6]. Psoriasiform syphilis is another atypical presentation of secondary syphilis that imitates psoriasis, and clinicians should be aware of this presentation to avoid the prescription of erroneous therapies [7]. In our patient, the simultaneous presence of the above-mentioned uncommon clinical manifestations made for a very intriguing case. Penicillin remains the gold standard treatment, and no cases of resistance to it have been described in the literature [8]. A single dose is sufficient to treat these atypical secondary forms. We observed a prompt response to treatment in our patient, and all his skin lesions healed within three weeks.

## Conclusions

This case report highlights the heterogeneity of skin lesions associated with syphilis: hyperkeratosis, psoriasis-like lesions, patchy alopecia, papules, and macules were observed in the same patient. Primary lesions, usually asymptomatic and self-resolving, can go unnoticed, and, not infrequently, a skin rash can be misleading. Palmoplantar lesions such as hyperkeratosis and moth-eaten alopecia are two signs suggestive of syphilis. Physicians should maintain a high index of suspicion for syphilis in all sexually active patients with a maculopapular rash or atypical skin manifestations. Syphilis should be excluded before starting steroids or other cutaneous treatment.
